# Integrating Treatment for Maternal Depression and Young Children’s Behavior Problems

**DOI:** 10.20900/jpbs.20230011

**Published:** 2023-12-27

**Authors:** Danielle Roubinov, Barbara Ivins, Laura Frame, Stephanie Simms, Linda Pfiffner

**Affiliations:** 1 Department of Psychiatry, University of North Carolina at Chapel Hill, Chapel Hill, NC 27514, USA; 2 Department of Psychiatry and Behavioral Sciences, University of California, San Francisco (UCSF), San Francisco, CA 94107, USA; 3 Early Intervention Services, Division of Behavioral Health, UCSF Benioff Children’s Hospital Oakland, Oakland, CA 94607, USA

**Keywords:** parental depression, child behavior problems, two generation, intervention, prevention

## Abstract

It is important to consider reciprocal associations between maternal and offspring mental health problems during early childhood. Existing interventions often focus narrowly on either adult or child mental health, missing the opportunity for holistic care. We describe the rationale and development of a pilot randomized clinical trial that explores their integration, combining an evidence-based parenting intervention with depression treatment to improve both maternal and child outcomes. Our approach is part of a growing field of two-generation interventions that offer a promising approach to enhance mental health support for caregivers and their young children.

## MATERNAL AND OFFSPRING MENTAL HEALTH ARE ASSOCIATED DURING EARLY CHILDHOOD

Depression among mothers is exceedingly common; approximately 1 out of every 10 offspring are exposed during childhood [1]. The COVID-19 pandemic created new parenting challenges and exacerbated existing stressors, leading to elevations in what were already concerning prevalence rates of maternal mental illness [2]. Due to the intertwined nature of early parent-child relationships, and the importance of parents as co-regulators of young children’s emotions, depression among mothers is detrimental not only for the impact that it has on morbidity and mortality for the diagnosed individual, but for the risks it poses to offspring health and development. Namely, children of parents with depression are at 3- to 6-fold increased risk for internalizing (e.g., sadness, fear, withdrawal) and externalizing (e.g., aggression, noncompliance, oppositionality) behavior problems [3–5]. Importantly, mutual transactions link parent and child mental health over time. There is a wealth of empirical literature demonstrating that maternal depression is prospectively associated with children’s behavior problems, and children’s behavior problems, in turn, prospectively heighten maternal depression [6–9]. Treatments that integrate care across generations are critical for simultaneously attending to maternal and child psychopathology [10]. Such intervention approaches are particularly important for mothers and their toddlers and preschool-age children: rates of maternal depression [11] and the associated risk of offspring maladjustment [12] are highly prevalent during this developmental period. While depression that emerges during the first postpartum year is a common target of treatment programs, that which occurs later (or chronically persists following childbirth) receives less explicit attention [13].

## THE NARROW AND SINGULAR FOCUS OF ADULT AND CHILD INTERVENTION PROGRAMS

Over two-thirds of adults with a mental health disorder are parents, yet depression treatments for adults generally address broad depressogenic cognitions and behaviors with little specific focus on parenting [10]. Adult depression interventions do not ameliorate negative parenting [14] nor do they consistently improve long-term child outcomes [15–17]. A singular focus similarly describes evidence-based treatments for young children’s behavior problems. Parenting-focused interventions target improvements in sensitive, nurturing, and responsive caregiving [18]. Despite robust associations between parent and child psychopathology [19], parenting interventions are not designed to treat parent psychopathology [20], nor have they proven sufficient for reducing parental depressive symptoms [21]. When the need arises to address parent mental health in the context of a parenting intervention, it is often handled with adjunctive referrals for adult psychotherapy or medication management with little regard for concurrent efforts to support parenting. Importantly, intervening to improve parental psychopathology may actually improve the efficacy of parenting interventions [22].

## INTEGRATING AN EVIDENCE-BASED PARENTING INTERVENTION WITH EMPIRICALLY-SUPPORTED DEPRESSION TREATMENT

There has been increasing interest in “two-generation” intervention approaches in recent years [23]. Important and impactful interventions have been developed to address the mental health and parenting needs of mothers, especially during infancy. For example, Ammerman and colleagues adapted cognitive behavioral therapy to be delivered in conjunction with standard home visiting programs for mothers during the first postpartum year (In-Home Cognitive Behavioral Therapy, IH-CBT; [24]. Relative to mothers who received standard home visiting only, those who received IH-CBT and ongoing home visiting reported lower levels of depressive symptom [25], though some research suggests the benefits may not extend to improvements in parenting or offspring outcomes [26]. In a study of family interventions for older children with disruptive behavior problems and their mothers with depression, the integration of cognitive therapy skills resulted in more sustained benefits on maternal mental health and child behavior than parenting skills alone [27]. A review of evidence-based interventions for mothers with depression and their young children highlights the salubrious effects of IH-CBT and Enhanced Triple P, the latter of which is an adaptation of the Triple P-Positive Parenting Program that includes supplemental modules focused on reducing parental depression and marital conflict [28].

Our team received support from the National Institute of Mental Health (NIMH R56MH127032; NCT05096611) to conduct a pilot effectiveness trial of a treatment designed to dually address the needs of mothers with depressive symptoms and toddlers/preschool-age offspring with behavior problems. Dyads were recruited on the basis of heightened maternal depressive symptoms and child behavior problems as determined by maternal report on validated rating scales. Mother-offspring dyads who enroll in our study are randomly assigned to receive *Attachment and Biobehavioral Catch-up (ABC)*, an empirically supported 10-session home visiting intervention that targets improvements in sensitive and responsive parenting [29] or *ABC*+*D*, an enhanced version of *ABC* that supplements the original *ABC* parenting content with brief videos to bolster mothers’ healthy coping and mood regulation skills. The “+*D*” adjuvant content is adapted from *Mothers and Babies*, an empirically-supported cognitive-behavioral intervention for maternal perinatal depression [30]. *ABC*+*D* is best conceptualized as a dual maternal depression-parenting intervention and importantly, this integrated intervention is more than the “sum of its parts.” *ABC*+*D* teaches mothers how skills for depression management and parenting are complementary. For example, the self-regulatory skills learned as part of treating depressive symptoms are also emphasized for their value in helping manage strong emotions during challenging interactions with children. The positive parent-child interactions fostered through *ABC* additionally attend to the anhedonic symptoms of depression.

**Figure 1.**
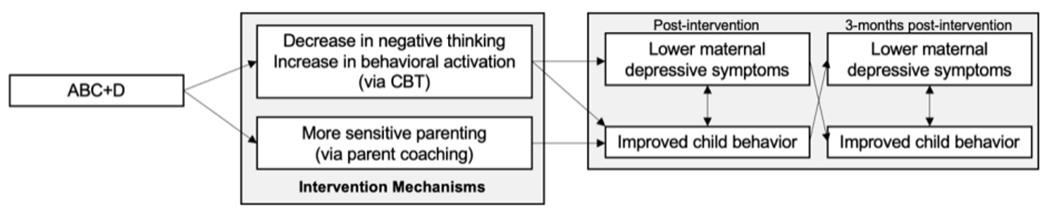
Conceptual model of ABC+D mechanisms and outcomes. Notes. *ABC*+*D* = Attachment and Biobehavioral Catch-up (ABC) plus cognitive-behavioral therapy-based video modules for maternal depressive symptoms. CBT = cognitive behavioral therapy.

Aligned with the NIMH experimental therapeutic approach [31], our study examines if *ABC*+*D* engages our proposed intervention mechanisms for mothers: lower levels of negative thinking and greater behavioral activation (via cognitive behavioral therapy-based skills) and greater parenting sensitivity (via *ABC* parent coaching), and if changes in these intervention targets are associated with lower maternal depression and child behavior problems. We compare *ABC*+*D* to *ABC* (the latter of which targets only parenting sensitivity), to understand which specific targets are essential for reducing the transactions between maternal depression and children’s behavior problems. Measures of maternal depression, child behavior problems, negative thinking, behavioral activation, and sensitive parenting are collected at pre-intervention and post-intervention. Additional assessments of maternal depression and child behavior problems are completed at 3-months post-intervention. Please see Figure 1.

As a pilot effectiveness study, the current randomized clinical trial is being implemented in a community setting. Our partner is Early Intervention Services, a multidisciplinary specialized program for children birth to 6 years old within the Division of Behavioral Health at the University of California, San Francisco (UCSF) Benioff Children’s Hospital Oakland. Recruitment for our study began during the height of the COVID-19 pandemic, and we encountered a number of practical challenges related to the public health crisis and implementation in a “real world” clinical environment. Many families preferred telehealth when the study launched, necessitating a rapid pivot to our plan for home visiting services. Importantly, *ABC* can be delivered with fidelity in a virtual format (i.e., TeleABC; [32]) and this approach has allowed us to deliver the intervention as intended. Tablets and high-speed internet hot spots are provided to families for whom technology would have served as a barrier to participation. Notably, many families continue to opt for virtual services even during less acute phases of the pandemic, improving access to care. We have worked to craft the most feasible delivery model for a combined focus on maternal mental health and parenting. During an initial rapid iteration phase of the study with four mother-child dyads, various “+*D*” delivery methods were trialed (e.g., immediately before, after, and separate from *ABC* parenting-focused content). The 45–60-min duration of ABC content and presence of offspring who understandably desired their mothers’ attention made fully combined depression and parenting intervention sessions challenging to deliver. Ultimately, it was determined that the 15-min “+*D*” sessions would be provided subsequent to each ABC session but on a different day (within one week of the corresponding *ABC* session and before the subsequent *ABC* session is delivered), allowing mothers brief, dedicated attention to their own mental health needs. This approach has increased feasibility of delivering the content, but requires a more intensive engagement for the intervention’s 10-week duration. Overall, it is important to recognize the substantial time and effort involved in interventions that integrate maternal depression treatment and parenting support. In the current study, mothers are additionally asked to complete study assessments, requiring time beyond treatment participation (though appropriate compensation is provided for completing outcome measures). The nature of parenting young children may pose challenges to extensive participation requirements, and our experienced clinical staff aim to be as accommodating as possible, including early morning, late afternoon, and rescheduled sessions as necessary. It is notable that the two aforementioned integrated programs are similarly intensive; IH-CBT is 15 weekly, 60-min sessions and a 1-month post-treatment booster session and Enhanced Triple P is 15 weekly, 60–90 min sessions. It will be important for future integrated interventions to explore innovative ways to balance the breadth of content needed to adequately address dual parental mental health and parenting concerns with the time and effort constraints inherent to the parental role.

Overall, integrated approaches to treating parent and child mental health concerns are promising and remain an area ripe for ongoing research. While outside the scope of the current viewpoint, our implementation in a community setting raised repeated questions about how best to simultaneously address families’ needs around social determinants of health and/or child developmental concerns, things not presently incorporated into the *ABC*+*D* model. It also raised important systems-level issues related to documentation, reimbursement, and billing for caregiver-focused services in a setting in which children are the “target” patient. California is approaching two-generation care in a transformative new way, implementing dyadic billing codes that offer sustainable funding for a short-term intervention like *ABC*+*D*, which aims to prevent more serious and long-term mental health problems for mothers and their young children. It is through efforts such as these that the promise of evidence-based two-generation approaches will be fully realized.

## Data Availability

Data collection is in progress. The dataset generated from this study will be made available in the National Institute of Mental Health Data Archive (NDA).
